# Strategies for Success: Simple Education Interventions to Equip Nursing Students in Rural Liberia

**DOI:** 10.5334/aogh.3251

**Published:** 2021-10-08

**Authors:** Daniel M. Maweu, Philip Davies, Lauretta Copeland Dahn, Viola M. Karanja, Merab Nyishime, Rosalita D. Rogers, Menkili G. Bindai, Rennie Viah, Helena L. Nuahn, Iona Thomas Connor, Joseph A. Verdier, Lydia W. Johnson, Rebecca Cook

**Affiliations:** 1 Partners In Health, Harper district, Maryland county, Liberia; 2 William V.S. Tubman University, Liberia

## Abstract

Severe shortages of skilled health workforce remain a major barrier to universal health coverage in low income countries including Liberia where nurses and midwives form more than 50% of the health workforce. According to the 2018 Service Availability and Readiness Assessment (SARA) report, Liberia has 10.7 core healthcare workers per 10,000 people, far below the WHO benchmark of 23/10,000 people. High quality training for nurses and midwives is one of the most important strategies to addressing these health workforce shortages. Since 2015, William V.S Tubman University (TU) faculty and Partners in Health (PIH) have partnered in nursing and midwifery education to address nursing and midwifery workforce shortages in Southeast Liberia. In our collaboration we have sought to not only increase the quantity of graduate nurses and midwives but also improve the quality of the training to ensure they are equipped to serve the population. TU strives to produce highly competent generic nurses who will excel in their clinical practice and future specialized training. By applying the theory of deliberate practice, learners are allowed to practice and self-evaluate repeatedly until they attain proficiency. Simulation training was adopted early in the training of nurses and midwives at TU to ensure students are well-prepared for real-life patient care. TU also established a preceptorship program to ensure that students receive skilled mentorship during clinical rotations. Internship for graduating senior Nursing/Midwifery students, where they focus on enhancing psychomotor and assessment skills, professional communication, safety and organization, medication administration and documentation, ensures successful integration into clinical practice after graduation. This progression of the student nurse or midwife from the exposure in the skills lab during pre-clinical modules, to individual preceptorship during clinical rotations to a structured internship experience with an intensive pre-internship “boot camp” have been the major innovations that have helped our partnership flourish. The foundation of these interventions is strong and sustained investment in nursing and midwifery faculty both at the university and the health facilities.

## Background

The world is currently facing an estimated shortfall of 18 million health workers needed to deliver and sustain universal health coverage by 2030 [1]. More than half of that shortfall is nurses and midwives. Liberia, still recovering from the impact of the 14-year civil war and the Ebola epidemic, has one of the lowest health workforce densities in the world. According to the World Health Organization (WHO)’s Global Health Observatory (GHO) data, Liberia had a total of 168 doctors and 453 nurses & midwives which translates to a ratio of 0.373 doctors & 1.007 nurses per every 10000 people in 2015 [2,3].

The main goal of nursing education is to prepare a nurse who can deliver safe, quality care that meet the diverse patients’ needs, function as transformational leaders, and advance evidence-based practice that benefits patients [4]. Nursing as a profession keeps evolving due to emerging diseases and advances in technology. As health systems adapt to these evolving disease burdens, nurses in Liberia are forced to take up more specialized responsibilities which previously were the reserved of doctors [5]. For instance, currently Non-communicable diseases (NCDs), such as cardiovascular diseases, cancers, and diabetes, contribute to 70% of death and 80% of disability in Liberia [6], nurses who were once primarily involved in the treatment of acute infectious diseases such as malaria are now also involved in assessing and treating patients with a range of complex chronic diseases.

**Figure 1:**
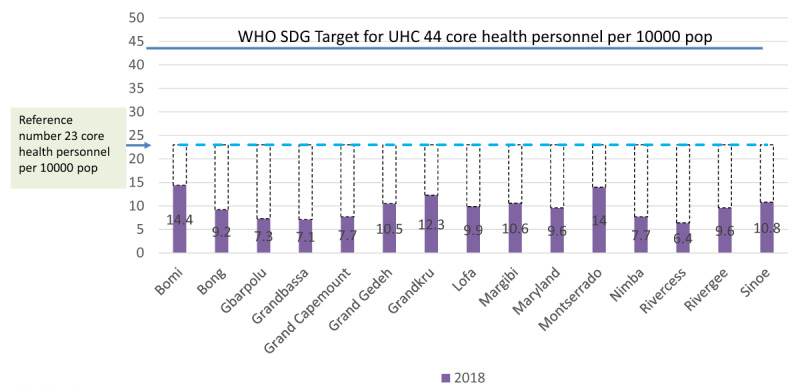
Liberia’s health workforce density by county – health professionals per 10,000 in 2018 [6,7].

Despite making significant strides in the post-war and post-Ebola period, Liberia is still struggling to rebuild the extensively destroyed healthcare system [7]. Health workforce gaps continue to be the worst in rural areas. Provision of basic Universal Healthcare coverage services in these settings is severely affected. According to Liberia Service Availability and Readiness Assessment (SARA) report 2018, Maryland county has a ratio of 9.6 core health worker per 10,000 people [8] which is below the minimum WHO recommendation of 22.8 doctors, nurses and midwives per 10 000 people [9] (***Figure 1**: Density of health workforce by counties – health professionals per 10,000*). In comparison, Montserrado county which hosts the capital city of Liberia, the density of core healthcare workers is 14 per 10,000 people [8]. This is still far below the WHO target for Sustainable Development Goal (SDG) of Universal Health Coverage (UHC), 44.5 per 10,000 [9].

TU department of nursing and midwifery was started in the year 2010 to address these shortages of nursing and midwifery workforce in the Southeast Liberia as the only university in as the only university in the region offering Bachelor of science degree in Nursing and midwifery. In 2015, PIH was invited by the Ministry of Health (MOH) to support the implementation of the national Investment Plan for Building a Resilient Health System, focusing specifically on JJ Dossen Memorial Hospital (JJD), Pleebo health center and TU in Maryland County. A firm foundation of the basic nursing training is a pre-requisite to further development of the advanced nursing specialists. TU is committed to ensure that our nursing and midwifery students receive the best training and maintain essential clinical competencies across all key areas of general patient care.

## Interventions to Strengthen Nursing and Midwifery Training in Rural Liberia

### Introduction of Bachelors of Science and Bridge programs in Nursing and Midwifery

One of the challenges identified in the Liberian health workforce is not simply the gaps in the absolute number of health professionals but also the level of training and experience. Many rural facilities are staffed by healthcare workers who have only completed a basic diploma in nursing (registered nurse) or certificate in midwifery (certified midwife). TU introduced a Bachelor of Science in Nursing (BSN) program in the year 2010 and accredited to offer Bachelor of science in Midwifery (BSM) in the year 2017. To meet the changes in demands for more skilled nursing workforce, higher levels of education and training of nurses are required through an improved education system that promotes seamless academic progression. To realize this goal, TU introduced Registered Nurse (RN) and Registered Midwife (RM) to bachelor’s degree bridging programs to give frontline nurses and midwives a chance for academic progression as they deliver healthcare services to the rural populations at their duty stations.

**Figure 2:**
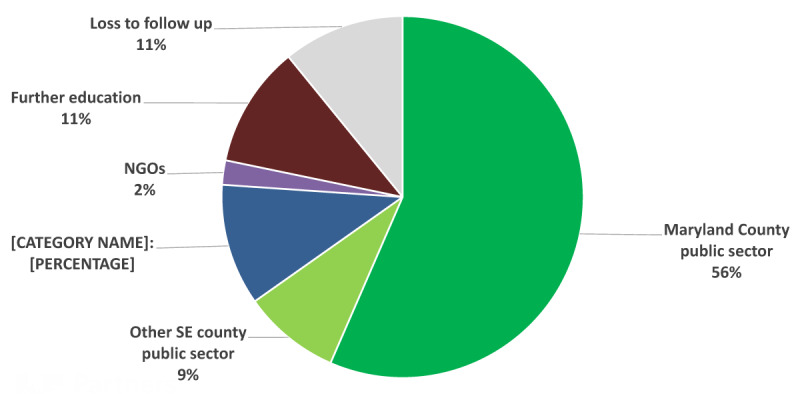
TU Nursing and midwifery graduate placement 2014–2017.

The bridge programs, especially that from certified to registered midwifery, is structured in a way to be flexible for midwives who are placed in rural or distant health facilities with didactic sessions once/week integrated with asynchronous learning assignments as well as an intensive skills workshops. To date a total of 181 BSc Nurses and five BSc Midwives have graduated from TU (***Table 1***: Accredited Nursing and Midwifery Training Programs offered at TU). Majority of these graduates are from the southeast part of Liberia with the rest coming from other regions within the country. A survey done by the university of nursing graduates from years 2014–2017 shows that 56% of the graduates from TU are currently employed by the MOH in Maryland county, 11% are employed by MOH in the remaining four counties in the south east Liberia, 11% proceeded for further education, 9% are employed by MOH in other counties within the country, 2% are employed by Non-Governmental Organizations (NGOs) and 11% were lost to follow. ***Figure 2*** TU Nursing and midwifery graduate placement 2014–2017.

**Table 1:** Nursing and Midwifery Training Programs offered at TU.


	BSN	RN-> BSCN BRIDGE	RM-> BSM

Year started	2010	2017	2018

Average enrollment/year	35	15	5

Total Graduates to-date	131	50	5


### Simulation Training

Simulation is an active pedagogical teaching strategy that helps students develop technical skills and critical thinking within a safe environment for students, their instructors and patients. It contributes to the training of competent nursing professionals [10]. Teaching and practicing invasive procedures on real life patients often poses ethical challenges in the clinical setting. As a result, nursing students should first be trained in a simulated, controlled and safe environment, to allow them make errors and learn from them without harmful consequences to patients [10]. Although it cannot replace real life clinical practice, simulation training has been essential at TU, in helping students transition to practice and also enabling teachers to transform from their pedagogic approach to teaching. The limited number of clinical placement and technological advancements have greatly contributed to the rise of simulation in nursing and midwifery training at TU.

TU has a simulation laboratory with basic and medium fidelity mannequins for use in training. The laboratory is coordinated by a TU lecturer highly proficient in both theory and practice. All nursing students undergo four months students undergo four months training in fundamentals of nursing skills at the lab before they qualify for clinical practice at the hospital. They undertake tasks designed to build their psychomotor skills and confidence before encountering real-life patients. The theory of deliberate practice is used extensively in this skills training. The theory posits that development of expertise requires incorporating a self-reflective feedback loop into the skill delivery or development (i.e., practice) process, rather than simply performing a task repetitively until mastered [11]. To achieve maximal efficiency, learners are allowed time to practice one skill in repeated cycles until they achieve mastery. The students use a skills training manual provided at the simulation laboratory for self-evaluation and also to ensure sequencing of the skills per procedure. At the end of the simulation laboratory training, the students are evaluated using an objective structured clinical examination (OSCE) which is compulsory for all to pass before they proceed for clinical practice.

JJ Dossen Memorial Hospital hosts more than 100 nursing students at various levels. These students often are crowded in any given clinical department, which can make it more difficult for students to get close supervision and coaching to practice and master some basic clinical skills if their only opportunity for skills refinement was on their clinical rotations.

The TU simulation training has assisted in ensuring that the students are well prepared to face the real patients in an environment with limited staffing. Some of the scenarios practiced in the sim lab, such as shoulder dystocia or breech extraction during childbirth, are rare to find at the hospital and thus simulation training is indispensable in such situations in equipping students with essential basic emergency obstetric and neonatal skills. This has helped the institution to prepare highly skilled nurses able to manage most medical and obstetric emergencies in remote areas before referral, filling in the vacuum of inadequate doctors. It is an important additional source of training along with the clinical settings as it produces a highly structured and well-defined environment for the student to learn.

### Developing Clinical Nurse Preceptors

To improve clinical mentorship of students at the clinical sites, TU recruited clinical preceptors from amongst the most proficient nurses from each department at the teaching hospital and health center. The students attend clinical practicums twice per week where they work under close supervision by the clinical preceptors in each department. Students require additional time and preparation to adequately meet their learning goals, which is practically impossible in low resource setting with current staff shortages. Without preceptors, these students would otherwise be left to work unsupervised to supplement the nursing shortages.

The students begin each shift with a pre-clinical conference attended by TU nursing lecturers and clinical preceptors where they discuss one disease/conditions they identified in the ward the previous day. The preceptorship model utilizes the theory of deliberate practice where students are allowed to observe a predetermined number of procedures being performed by the preceptor or by a licensed nurse before they attempt to perform them first under observation and then independently. Depending on the level of skill required, each procedure has preset minimum number of expected attempts as per their logbooks under observation until the learner is proficient.

To motivate these clinical preceptors, a monthly stipend is given to them. They also attend an annual one-day preceptorship faculty development workshop to keep them abreast with the current trends in nursing education. This ongoing preceptor development is critical in ensuring that preceptors are well-equipped to provide adequate supervision and mentorship.

### Improving Nursing and Midwifery Student Internship through focused pre-deployment skills-building

Clinical internship for nursing students is an important approach to bridge the gap between training and professional life [12]. Internship occurs during the final year of training. The students are sent to different referral hospitals within Southeast Liberia for their three-month internship program. To prepare these prospective nurses for the clinical practice during internship and post-graduation/licensure, the students undergo a three week long pre-internship training. During this training, they are taken through Basic Life Saving Skills (BLSS) in maternal and newborn health, Integrated Management of Childhood Illnesses (IMCI) short course and basic Infection prevention and control (IPC) practices. The affiliation programs have many benefits for students since they get a chance to engage in real-life clinical situations with less supervision, understand workplace dynamics and the basic ward administration tasks. It initiates adaptability and creativity, provides greater opportunity to learn clinical skills, and eventually prepare the student for the future job experience. However, without the pre-internship workshops where students are equipped with skills and resources, internship experiences could be morally distressing as students might find themselves with limited supervision and support. The feedback from the students has been that the training prior to internship is a key contributing factor to the success of their career as nurses.

## Themes and Recommendations

### Deliberative Practice

Clinical training of nurses and midwives in our education partnership at the Southeast Liberia, is heavily based on the theory of deliberative practice. This method of clinical teaching was adopted following feedback from TU graduate nurses and their employers that the clinical teaching did not prepare them adequately for the clinical practice. Previously the students were left under the mentorship of nurses in the clinical practice area without any structured learning guide. Currently, the students are given feedback continuously during each practice session and summative feedback at the end of every semester. The student’s express satisfaction with this approach since it allows them an opportunity to observe the procedure first, then repeat it under supervision with both self-evaluation and evaluation from preceptors. Following their internship, they’ve verbalized confidence with their level of skills as they transition to nursing practice. We believe that nursing and midwifery education, especially clinical training, should be structured to incorporate deliberative practice to equip graduate nurses and midwives with the right skills competency.

### Investment in Faculty

The recommended teacher to student ratio by the Liberia Board of Nursing and Midwifery (LBNM) for classroom instruction and clinical instruction is 1:50 and 1:10 respectively [13]. The importance of faculty recruitment, retention and development has been a key concern at the TU nursing department. The department is currently understaffed with only five faculty teaching more than 120 students. This leads to fatigue and reduction of opportunities to interact with the students at their clinical sites. Recently three faculty have been sponsored to complete their master’s in nursing education which will in the long-term greatly strengthen the nursing department, however it does require careful planning and adequate funding to support short-term faculty gaps. More options for robust, contextually-appropriate, and affordable asynchronous masters and faculty development programs are important priorities. In addition, there is early promise of specialized nursing career pathways to strengthen patient care which could be offered as post-graduate diplomas or masters. We recommend the expansion of nursing training at TU, the further development of TU faculty, and the development of advanced nursing pathways in the LBNM to embrace the clinical demand of nursing specialists in key areas like critical care, neonatal care, and oncology. To build a sustainable nursing specialist programs in Liberia, there is need for international nursing specialist scholarship programs. This will ensure an optimal number of nurses travel for specialist training in other countries especially within Africa after which they will come back and start specialized training in Liberia.

### Continuous Curriculum Review and Reform

Continuous evaluation of a new curriculum during its implementation is important. Students, faculty, stakeholders and alumni note that the current BSc. curriculum focuses 18 months to basic general university courses leaving only 30 months to focus on nursing courses. This could be restructured to dedicate more time to the nursing courses as compared to the general university courses to improve the quality of the nurses graduating from the program. There is a need for close collaboration between the national nursing and midwifery boards, nursing faculty, trainees and ultimately feedback from patients to ensure that pre-service nursing and midwifery curriculum is meeting the goals of developing a “fit-for-purpose, productive, and motivated health workforce” as envisioned in the health workforce strategy [7]. While OSCEs are currently in use to evaluate nursing and midwifery students in the skills lab, there is no provision for OSCE of students during their clinical training or licensing exams.

## Conclusion

With the changes in disease trends and continued health workforce gaps, Liberia is in need of well-trained nursing and midwifery practitioners. The quality of preservice nursing/midwifery training has a direct impact to the quality of care given to patients. The number of faculty per student ratio has a direct impact to the quality of skills gained by nursing students. There’s need for investing in specialized training of nurses in low resource settings to increase the numbers of nursing faculty. Simulation lab training does not replace clinical training of students but serves as an equally important part of training in low resource settings where nurses are expected to take up more specialized responsibilities due to shortage of doctors. Simulation training is especially effective when combined with deliberative practice and preceptorship in clinical rotations. Conducting robust pre-internship training course has demonstrated value in successfully launching senior students into their internships and early careers with more confidence and competence. Strategic investment in nursing and midwifery faculty, including advanced training in education and nursing specialization pathways are important strategies to address the current health workforce challenges in Liberia.

## Figures and Tables

**Table:** 

GHO:	World Health Organization (WHO)’s Global Health Observatory
PIH/L:	Partners in Health Liberia
MOH:	Ministry of Health
TU:	William V.S Tubman University
NCDs:	Non-Communicable Diseases
LBNM:	Liberia Board of Nursing and Midwifery
NCSBN:	National Council of State Boards of Nursing
IMCI:	Integrated management of childhood Illnesses
IPC:	Infection prevention and control
PITC:	Provider Initiated Testing and Counselling
HIV:	Human Immunodeficiency Virus
ART:	Antiretroviral Therapy
BSN:	Bachelor of Science in Nursing
BSM:	Bachelor of Science in Midwifery
OSCE:	Objective Structured Clinical Examination
SARA:	Service Availability and Readiness Assessment
